# Evaluating the Impact of Criminalizing Drunk Driving on Road-Traffic Injuries in Guangzhou, China: A Time-Series Study

**DOI:** 10.2188/jea.JE20140103

**Published:** 2016-08-05

**Authors:** Ang Zhao, Renjie Chen, Yongqing Qi, Ailan Chen, Xinyu Chen, Zijing Liang, Jianjun Ye, Qing Liang, Duanqiang Guo, Wanglin Li, Shuangming Li, Haidong Kan

**Affiliations:** 1 School of Public Health, Key Lab of Public Health Safety of the Ministry of Education, & Key Lab of Health Technology Assessment of the Ministry of Health, Fudan University, Shanghai, China; 2 Environmental & Occupational Health Evaluation Department, Shanghai Municipal Center for Disease Control & Prevention, Shanghai, China; 3 Global Health Institute, Fudan University, Shanghai, China; 4 Guangzhou First-Aid Service Command Center, Guangzhou, China; 5 Department of Cardiology, The First Affiliated Hospital of Guangzhou Medical University, Guangzhou, China; 6 State Key Laboratory of Respiratory Disease, Guangzhou Institute of Respiratory Disease, The First Affiliated Hospital, Guangzhou Medical University, Guangzhou, China; 7 Guangzhou Hoffmann Institute of Immunology, School of Basic Sciences, Guangzhou Medical University, Guangzhou, China; 8 Department of Pathogenic Biology, Guangzhou Medical University, Guangzhou, China; 9 Department of Emergency, The First Affiliated Hospital of Guangzhou Medical University, Guangzhou, China; 10 Department of Gastrointestinal Surgery, Affiliated Guangzhou First Municipal People’s Hospital, Guangzhou Medical University, Guangzhou, China

**Keywords:** road-traffic injuries, drunk driving, time series

## Abstract

**Background:**

Road-traffic injury (RTI) is a major public-health concern worldwide. However, the effectiveness of laws criminalizing drunk driving on the improvement of road safety in China is not known.

**Methods:**

We collected daily aggregate data on RTIs from the Guangzhou First-Aid Service Command Center from 2009 to 2012. We performed an interrupted time-series analysis to evaluate the change in daily RTIs before (January 1, 2009, to April 30, 2011) and after (May 1, 2011, to December 31, 2012) the criminalization of drunk driving. We evaluated the impact of the intervention on RTIs using the overdispersed generalized additive model after adjusting for temporal trends, seasonality, day of the week, and holidays. Daytime/Nighttime RTIs, alcoholism, and non-traffic injuries were analyzed as comparison groups using the same model.

**Results:**

From January 1, 2009, to December 31, 2012, we identified a total of 54 887 RTIs. The standardized daily number of RTIs was almost stable in the pre-intervention period but decreased gradually in the post-intervention period. After the intervention, the standardized daily RTIs decreased 9.6% (95% confidence interval [CI], 6.5%–12.8%). There were similar decreases for the daily daytime and nighttime RTIs. In contrast, the standardized daily cases of alcoholism increased 38.8% (95% CI, 35.1%–42.4%), and daily non-traffic injuries increased 3.6% (95% CI, 1.4%–5.8%).

**Conclusions:**

This time-series study provides scientific evidence suggesting that the criminalization of drunk driving from May 1, 2011, may have led to moderate reductions in RTIs in Guangzhou, China.

## INTRODUCTION

Road-traffic injury (RTI) is a major but commonly neglected public-health concern worldwide. In 2004, the World Health Organization (WHO) estimated that 1.2 million people are killed each year in road crashes worldwide, while the number injured could be as high as 50 million. The number of RTIs, if no additional robust preventions were implemented, was projected to increase by almost 65% between 2000 and 2020 and by as much as 80% in low- and middle-income countries.^1^ As the largest developing country, China is undergoing rapid urbanization and motorization. China is experiencing a disproportionately high burden of RTIs relative to its level of motorization. According to the results of the 3rd National Retrospective Survey on Causes of Death, the road-traffic-related fatality rate was 15.3 per 100 000 population in 2005, indicating that almost 200 000 Chinese people were killed in that year as a result of road-traffic collisions.

A sound body of scientific evidence indicates that drunk driving increases the risk of being involved in traffic crashes, as well as the severity of resulting injuries.^2^^–^^4^ There are no national statistics available on drunk driving in China. A preliminary survey in two southern cities from 2006 to 2009 showed that 6.9% of the drivers had a non-zero blood-alcohol concentration (BAC) and 4.6% had a BAC higher than the maximum legal limit of 20 mg/dL. In this survey, 25.7% of all serious crashes (in which one or more victims were hospitalized) and 48% of all fatal crashes were found to be alcohol-related.^5^

Stringent laws and regulations have been introduced in some countries to reduce the rate of drunk driving and have been shown to be effective in decreasing rate of alcohol-related RTIs.^3^^,^^6^^–^^10^ Given the substantial contribution of alcohol to RTIs, since 2004, the Chinese government has adopted increasingly stricter policies and laws to combat drunk driving.^5^ The landmark 8th Amendment to the Criminal Code enacted by the National People’s Congress criminalized drunk driving beginning on May 1, 2011. The 8th Amendment to the Criminal Code was the sole key intervention to combat drunk driving in China, where the situation of drunk driving was severe. However, no studies have been conducted to evaluate the effectiveness of this intervention. Therefore, we conducted this interrupted time-series analysis to evaluate the impact of criminalizing drunk driving on road-traffic injuries in Guangzhou, China.

## METHODS

### Study settings

According to the amended law, those who drive a motor vehicle with a BAC between 20 and 79 mg/dL will be punished by the public-security bureau, including suspension of the driver’s license for at least 6 months and a fine of 1000–2000 CNY (approximately $160–320); further, if they drive a commercial vehicle, a detention of 15 days and a fine of 5000 CNY will be added. Those who drink and drive with a BAC greater than 80 mg/dL will lose their license for at least 5 years and will face prosecution for criminal offenses. Prior to this intervention, the inspection of driving after drinking was rare and the punishment was mild.

**Figure 1.**
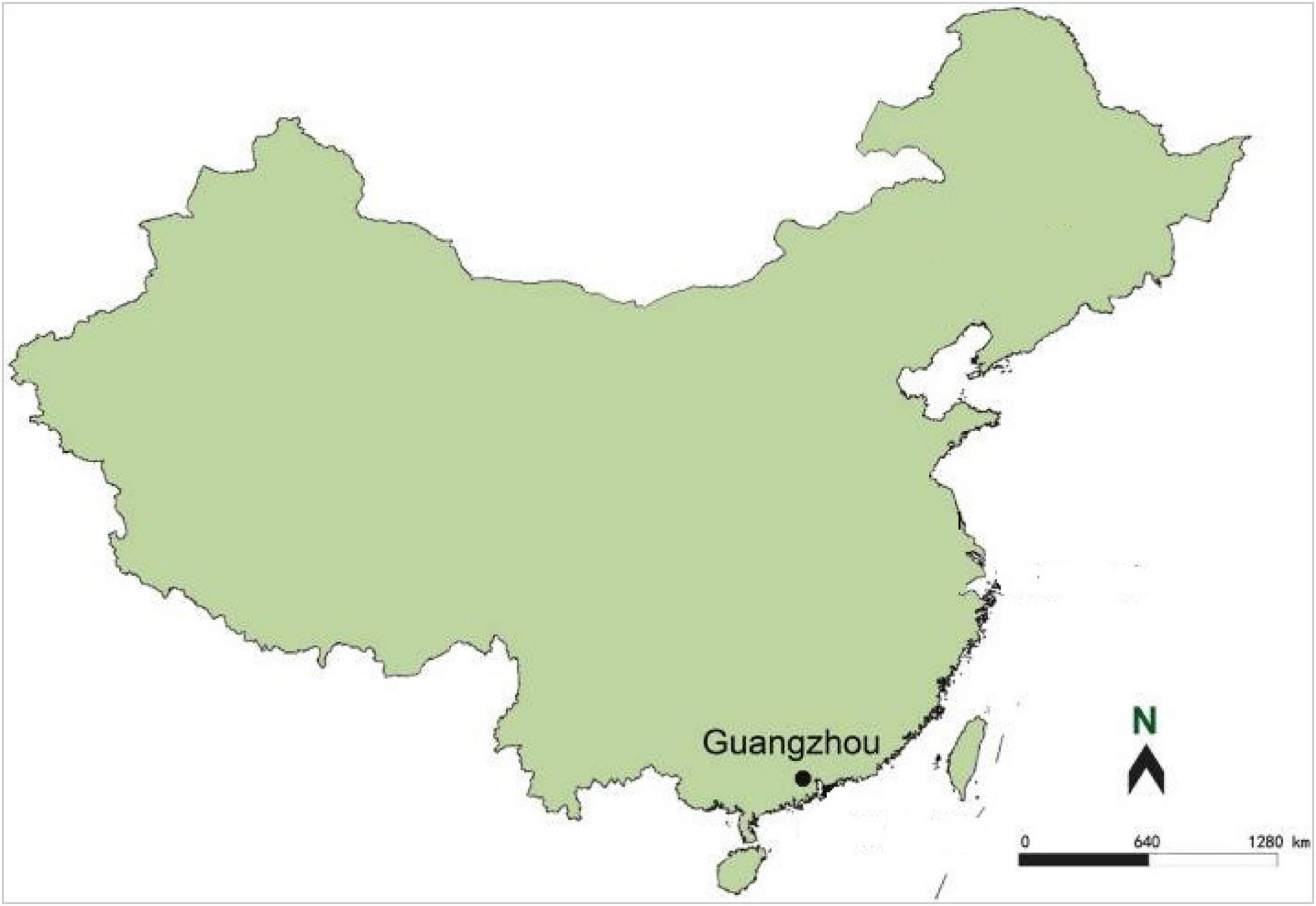
Location of Guangzhou City in China.

As the provincial capital of Guangdong Province, Guangzhou is located in the southern part of Mainland China (Figure 1). Since the reform and opening-up policy in 1978, its GDP has grown at a double-digit rate every year, and Guangzhou has continuously ranked first in growth among all cities in the country. The number of motor vehicles in Guangzhou reached 2 million in 2012.

According to the statistics published by the Ministry of Public Security, after 2 years of law enforcement beginning on May 1, 2011, 870 000 cases of driving after drinking alcohol, including 120 000 cases of drunk driving (BAC greater than 80 mg/dL), had been punished across China. In 76% of these cases, drunk drivers were transferred to procuratorial authorities, and more than 57% of drunk drivers were judged by the courts.^11^ In Guangzhou, 586 cases of drunk driving were transferred to procuratorial authorities, and 84% of these had been sentenced 1 year after the intervention.^12^

### Data collection

We collected daily aggregate data on RTIs from the Guangzhou First-Aid Service Command Center (GFASCC) from 2009 to 2012 according to the ambulance attendance records. The GFASCC is the sole public ambulance service system in Guangzhou, and no ambulance cars are operated by individuals or non-governmental organizations. It covers more than 7 million people in the urban areas of Guangzhou. The causes of ambulance attendances were determined by physicians in the ambulance cars. We defined RTIs as all injuries that resulted from a road crash and required transport by ambulance, irrespective of whether the patient survived, so RTIs included drivers, passengers, and pedestrians. Off-road injuries, such as those from garage, farmland, and railway crashes, were not included in this study.

To allow for standardization and comparability, we also collected the annual number of motor vehicles and population in this city from the Guangzhou Statistics Bureau. Monthly and daily data of this kind were not available.

Because a previous study in Guangdong Province showed that drunk driving was more prevalent at night,^13^ we divided the daily total RTIs into nighttime (8:00 pm to 6:59 am) and daytime (7:00 am to 7:59 pm) RTIs, and we also standardized rates of RTIs per 1 million population and 1 million vehicles. We also collected the daily number of alcohol-related and non-traffic injuries from GFASCC to examine whether there was a change before and after the intervention.

Data were analyzed at the aggregate level, and no individual records/information for patients were involved, so this study received a waiver for informed consent. Additionally, as no participants were contacted, written informed consent was not obtained. This study does not involve experimental animals or individual information on human subjects. The protocol of this study was approved by the Institutional Review Board of the School of Public Health, Fudan University.

### Statistical analyses

Guangzhou has long been one of the most crowded cities in China, with numbers of vehicles and residents increasing in recent years, so it was inappropriate to directly analyze the crude data on daily RTIs. Considering the rapid expansion on vehicles and population compared with the slow increase of road area during our study period, it was plausible to assume that both the population size and number of vehicles might contribute to the risk of RTIs. To compare rates of RTIs in different years, we standardized the crude RTIs into rates per 1 million population and 1 million vehicles. We then calculated the monthly average change in standardized daily RTIs before (January 1, 2009, to April 30, 2011) and after (May 1, 2011, to December 31, 2012) the intervention (ie, the criminalization of drunk driving on May 1, 2011) and assessed the statistical significance by a *t* test of the means.

Daily RTIs can change with time because of secular changes in social norms, personal habits, or other factors. We therefore evaluated the impact of criminalizing drunk driving on RTIs using an interrupted time-series design.^14^^,^^15^ This approach has the advantage of automatically controlling time-invariant confounders by examining the same population repeatedly over time. Time-series designs make use of a large series of observations on a daily basis and are widely used in public-health studies to evaluate the short-term effect of a change in the environment, such as air pollution or ambient temperature.^16^^–^^18^ Because daily RTIs approximately follow a quasi-Poisson distribution (ie, the variance is greater than the mean),^19^ we applied the overdispersed generalized additive model to explore the association of RTIs and the explanatory variable.^20^

For the main model, we introduced a dummy explanatory variable “intervention” to compare the standardized daily rates of RTIs before (January 1, 2009, to April 30, 2011) and after (May 1, 2011, to December 31, 2012) the intervention. We included five time components as covariates: (1) calendar day, to adjust for long-term trends; (2) a pair of sine and cosine terms, to model one cycle per year to adjust for seasonal patterns; (3) an indicator variable for the day of the week, to account for within-week variation; (4) a dummy variable for “public holidays”; and (5) an interaction term, to take into account the differences in the temporal trend before and after the intervention.^21^ The model formula can be summarized as follows:

$$\begin{array}{l} \text{ln}[\text{E}(Y_{t})]=\beta_{0}+\beta_{1}X_{t}+\beta_{2}\text{t}+\sum_{i=1}^{i}[\text{sine}(\beta_{3})+\text{cosine}(\beta_{4})]\\ \;+\sum_{w=0}^{w}\beta_{5}DOW_{t}+\beta_{6}Holiday+\beta_{7}X_{t}\text{t}+\beta_{8}\varepsilon \end{array}$$

where t is the time period (eg, t = 1 for the first day of the series, t = 2 for the second); X_t_ identifies the pre- and post-intervention periods (X_t_ = 1 for the post-intervention period, 0 for the pre-intervention period); i takes values of years between 1 and 4 (eg, k = 1 for the first year, k = 2 for second year); sine is the sine function; cosine is the cosine function; DOW is the indicator of the day of the week (eg, w = 0 for Sunday and 1 for Monday); Holiday is the binary dummy variable (1 for public holidays, 0 for non-holidays); X_t_ t is the dummy variable for the intervention, multiplied by the time trend (t) (ie, an interaction term) to take into account the differences in the time trend before and after the intervention; and ε is the error term. We derived the coefficient (β_1_) and its standard error of the “intervention” variable from the main model and computed the percentage change and its 95% confidence interval (CI) of standardized daily RTIs after and before the intervention. To our knowledge, there were no other important road-safety laws introduced during the study period.

We replaced daily RTIs with daily nighttime RTIs and daily daytime RTIs in the same model, to see whether there was a change before and after the intervention. We selected the daily number of non-traffic injuries and alcoholics from the same ambulance system as an indicator that was not logically related with the intervention, which was then introduced into the same model with daily RTIs as the dependent variable after standardization by 1 million population.

We performed three sensitivity analyses to evaluate the robustness of our results to statistical models. First, we used another definition of nighttime (6:00 pm to 5:59 am) and daytime (6:00 am to 5:59 pm) in the same models. Second, we fit the time-series models using monthly rates of RTIs, nighttime/daytime RTIs, alcoholism, and non-traffic injuries, in which calendar day (*t* in the formula) was replaced with “month” from 1 to the end, and the “day-of-week” and holiday variables were removed. Third, we used 2 and 3 pairs of sine and cosine terms per year for seasonality control.^22^

The statistical tests were two-sided, and values of *P* < 0.05 were considered statistically significant. All models were fitted in R (version 2.15.1; R Foundation for Statistical Computing, Vienna, Austria) with the GAM using the “*mgcv*” package.

## RESULTS

Table 1 shows the basic descriptive information in this study. From January 1, 2009, to December 31, 2012, we identified a total of 54 887 RTIs. The crude daily RTIs increased gradually from 2009 to 2012, along with the increasing number of motor vehicles and the growing population. During the study period, we identified a total of 19 521 nighttime RTIs and 35 366 daytime RTIs. Table 1 shows that the standardized RTIs and nighttime/daytime RTIs decreased during the year 2011. Table 1 also indicates that the daily counts of alcoholism and non-traffic injuries increased during the study period, from 2009 through 2012.

**Table 1.** Basic descriptive statistics in this study

Variables	2009	2010	2011	2012
Daily mean RTIs	31.3	38.6	41.6	41.7
Annual number of vehicles (×1 million)^a^	1.3	1.6	1.9	2.0
Annual population (×1 million)^a^	7.9	8.0	8.1	8.2
Standardized daily RTIs^b^	3.0	3.0	2.8	2.5
Standardized daily daytime RTIs	1.9	1.9	1.8	1.6
Standardized daily nighttime RTIs	1.1	1.1	1.0	0.9
Daily mean cases of alcoholism	13.3	17.4	21.0	24.8
Daily mean non-traffic injuries	44.1	50.0	49.2	50.6
Daily alcoholism per 1 million population	1.7	2.2	2.6	3.0
Daily non-traffic injuries per 1 million population	5.6	6.2	6.0	6.2

RTIs, road traffic injuries.

^a^Data source: Guangzhou Statistical Bureau.

^b^RTIs were standardized as a unit of 1 million population and 1 million vehicles.

**Figure 2.**
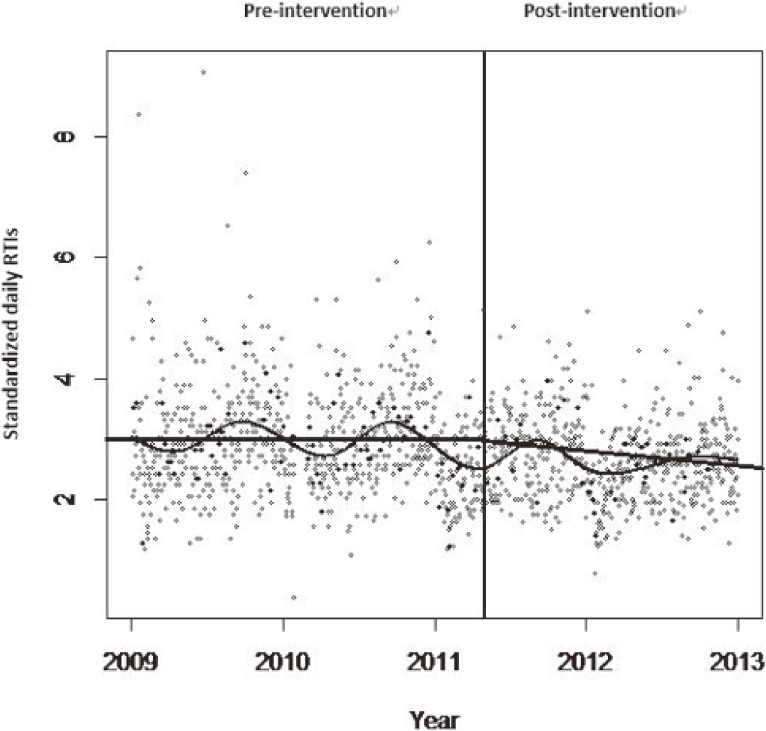
The time series of standardized daily RTIs in Guangzhou, 2009–2012. Daily RTIs are standardized by a unit of 1 million population and 1 million vehicles. The vertical line indicates the enforcement of the law on May 1, 2011, in China. The curve represents the smooth lines of RTIs against the date (natural spline with 4 degrees of freedom per year). The straight lines represent the slopes before and after the intervention. The X-axis represents the calendar day during the study period. The Y-axis represents the number of standardized daily RTIs. RTI, road traffic injuries.

Figure 2 illustrates that the standardized daily RTIs varied over time, with an apparent peak in the fall. Figure 2 also suggests that the standardized daily RTIs were almost stable in the pre-intervention period but decreased gradually in the post-intervention period. There were similar decreasing trends of daily daytime and nighttime RTIs.

Table 2 shows decreases in the monthly totals of standardized RTIs after the intervention. On average, standardized RTIs declined 12.4%, with the largest decreases in fall. Table 2 also shows that the monthly differences between the pre-intervention and post-intervention period were statistically significant.

**Table 2.** Monthly average difference in standardized RTIs^a^ before and after intervention in Guangzhou, China

Month	Pre-intervention	Post-intervention	Difference	*P* value^b^
Jan.	81	69	12	<0.001
Feb.	67	61	6	0.006
Mar.	85	75	10	<0.001
Apr.	80	76	4	0.009
May	97	82	15	<0.001
Jun.	86	82	4	0.008
Jul.	91	84	7	<0.001
Aug.	96	82	14	0.008
Sep.	94	84	10	<0.001
Oct.	107	84	23	<0.001
Nov.	97	89	8	<0.001
Dec.	103	82	21	<0.001
Total	1085	951	134	<0.001

RTIs, road-traffic injuries.

^a^RTIs were standardized as a unit of 1 million population and 1 million vehicles.

^b^The differences were examined by *t* test on a daily basis.

In the main models, the coefficient β_7_ of the intervention was 0.096 (95% CI, 0.065–0.013). Correspondingly, we estimated a 9.6% (95% CI, 6.5%–12.8%) decrease in standardized daily RTIs after the intervention (see Table 3). The slope coefficient of the temporal trend in the pre-intervention period was small and statistically insignificant (*P* = 0.325); however, the slope decrease was statistically significant in the post-intervention period (*P* = 0.004).

**Table 3.** Mean percentage change and slope coefficients of standardized daily and monthly counts of road-traffic injuries, alcoholism, and non-traffic injuries before and after intervention in Guangzhou, China

	Daily counts			Monthly counts		
	
	% change (95% CI)	Pre-slope (%, *P* value)	Post-slope (%, *P* value)	% change (95% CI)	Pre-slope (%, *P* value)	Post-slope (%, *P* value)
Road-traffic injuries^a^	−9.6 (−12.8, −6.5)	−0.002 (0.325)	−0.006 (0.004)	−11.9 (−19.7, −4.0)	−0.13 (0.67)	−0.36 (0.03)
Alcoholism^b^	38.8 (35.1, 42.4)	0.060 (<0.001)	0.057 (<0.001)	40.1 (28.4, 51.8)	1.79 (<0.001)	1.71 (<0.001)
Non-traffic injuries^b^	3.6 (1.4, 5.8)	0.009 (0.003)	0.006 (<0.001)	2.8 (−3.5, 9.1)	0.32 (0.22)	0.21 (0.10)

CI, confidence interval.

^a^Road-traffic-related injuries were standardized as a unit of 1 million population and 1 million vehicles.

^b^Alcoholism and non-traffic-related injuries were standardized as a unit of 1 million population. These estimates were obtained from the generalized additive model described in the “Statistical analysis” section.

We found a 13.3% (95% CI, 7.2%–19.3%) decrease in standardized daily nighttime RTIs after the intervention; the corresponding decrease was 6.5% (95% CI, 5.8%–13.4%) in standardized daily daytime RTIs. In comparison, the standardized daily alcoholism increased 38.8% (95% CI, 35.1%–42.4%), and non-traffic injuries increased 3.6% (95% CI, 1.4%–5.8%) (see Table 3).

In the sensitivity analyses, we estimated 11.6% and 8.6% decreases of RTIs in nighttime and daytime, respectively, using alternative definitions. The estimated changes in RTIs (both daytime and nighttime), alcoholism, and non-traffic injuries on a monthly basis were similar to those on a daily basis, but the CIs were wider (Table 3). Our results did not vary substantially after controlling for seasonality (data not shown).

## DISCUSSION

Drunk driving is one of the major risk factors for road safety worldwide. This study in Guangzhou, China suggested that the criminalization of drunk driving on May 1, 2011 was followed by a statistically significant reduction in the number of standardized daily RTIs, despite increases in the standardized daily rates of alcoholism and non-traffic injuries during the same period. Our analysis may, at least to some extent, suggest that rigorous control of alcohol-impaired driving (eg, criminalization) could confer benefits in improving road safety.

Previous studies have found increasing enforcement has a large effect on road safety and is cost-effective.^3^^,^^19^^,^^23^ In this study, although progress has been made to improve traffic safety, the number of road injuries did not decrease in Guangzhou from 2009 to 2010 (before the enactment of the 8th Amendment to the Criminal Code), regardless of whether the data were standardized for the increase in motor vehicles and population. On May 1, 2011, a more stringent law criminalizing drunk driving was implemented. We evaluated its effects on the decrease in the number of victims of traffic crashes using a time-series design. Our analysis indicated a moderate but statistically significant impact of criminalizing drunk driving on RTIs. We found that the criminalization of drunk driving may have prevented 9.6% of RTIs, and thus demonstrated that this legislation was a successful public-health intervention. In this study, we also found a 13.3% decrease in standardized daily nighttime RTIs after the intervention, with a corresponding decrease of 6.5% in standardized daily daytime RTIs. The difference was probably due to the high prevalence of drunk driving at night. The lessons learned from this intervention in China might be useful for other countries attempting to reduce rates of injuries associated with drinking and driving.

Many policy interventions have been demonstrated to reduce traffic fatalities and injuries in the scientific literature, such as use of seat belts,^24^ criminalizing road-traffic offenses,^19^ and installation of speed cameras.^14^^,^^25^ To our knowledge, China is to date one of the few countries to have criminalized drunk driving; the effects of this policy intervention vary in the scientific literature. In Spain, several investigators have reported that the effect of criminalizing drunk driving ranged from no impact to a 73% reduction in the number of alcohol-related collisions.^15^^,^^19^^,^^25^ In Canada, the enactment of the criminal law specifying the legal BAC was followed by an 18% decrease in the number of fatally injured drunk drivers; no corresponding effect was observed for non-drunk-driver fatalities.^6^ A study in Taiwan showed that criminal sanctions for drunk driving reduced the expected number of fatal drunk driving crashes on average by 72.6% over 20 months following their implementation.^7^ Since the criminalization of drunk driving in the United States, researchers have found 6%, 5%, and even 0% reductions in the number of alcohol-related road fatalities in different studies.^26^^–^^28^ An early time-series analysis in Norway and Sweden found that traffic deaths were reduced simultaneously with legal reforms that included abandonment of mandatory jail sentences for persons driving with BACs above specific limits.^29^ A recent study in Thailand suggested that, compared with doing nothing, comprehensive intervention including mass-media campaigns, random breath testing, and selective breath testing are all cost saving and have the potential to reduce the burden of alcohol-related road-traffic injuries by 24%.^30^

As the intervention was nationwide, other cities were not used as control groups. The time-series design can automatically control for time-invariant confounders and adjust for temporal trends, seasonality, and day of the week, which may influence the rate of RTIs. We examined the change before and after the intervention using dependent variables that were not logically related to this intervention. Using the same models with RTIs, we found that cases of alcoholism increased by 38.8%. This change was supported by the data from “China Monthly Economic Indicators” published by the National Bureau of Statistics, which showed a mean annual increase of 43.1% in the consumption of white spirits and 11.8% in the consumption of beer in 2011–2012 compared to 2009–2010. This additional analysis also offers another perspective: RTIs were likely to increase significantly in the context of the high rise in alcoholism if the intervention was not implemented. The results suggest that the intervention can lead to moderate reductions in road injuries in the context of rapid increases in the consumption of white spirits and alcoholism in China. Furthermore, we did not find a substantial increase in non-traffic injuries after the intervention (the increase in non-traffic injuries was statistically significant on a daily basis but not on a monthly basis). Therefore, our findings were not likely obtained by chance or by the simultaneous decreasing trends of alcohol drinking and all types of injuries.

A previous study showed that the onset of a declining trend in fatal motor vehicle crashes involving drunk driving might be merely caused by changes in drunk-driving behavior after a high-profile fatal crash.^31^ After an initial review of the reports on high-profile fatal crashes in China, there were no such cases around the time of starting the law enforcement. Therefore, it is not necessary to consider this possible pre-law change.

In addition to the time-series design, another advantage of this study was the use of data from GFASCC. Currently, there are two other available official sets of statistics for road crashes and casualties in China. The first set is collected by the Traffic Administrative Bureau under the Ministry of Public Security, which aggregates the summary records of all investigated road crashes and causalities. Road crashes might have been underreported in the police-reported data collected by traffic administrative agencies because they happened outside the public road jurisdiction and because a new performance index for measuring safety at work may have influenced reporting to meet annual reduction targets in China. In contrast, the GFASCC received almost all traffic-related injuries and did not have any performance index for a reduction target. A previous study showed that the number of traffic-related deaths reported by health departments was almost twice the number reported by traffic police department.^32^ Therefore, data from the GFASCC were more reliable.^33^ The second source is the National Disease Surveillance System, which is run by the Chinese Center for Disease Control and Prevention. However, this system currently covers approximately 6% of the total population and issues annual (but not daily) reports on causes of deaths, including traffic crashes.

Several limitations to the present study should be addressed. First, as we were limited by the availability of data, we were not able to estimate the number of RTIs attributable to drunk driving nor obtain detailed information about how many drivers, passengers, and pedestrians were involved in alcohol-related road crashes. Second, kilometers traveled by vehicle may be a better exposure denominator for the standardization of daily RTIs; however, these data were not available. Third, monthly or daily standardization of RTIs was also impractical because only annual data on numbers of vehicles and population were available. All of these limitations were due to lack of some important data (actually these data were not routinely collected in China), so the magnitude and directions of the influences of these limitations were complicated and difficult to determine. Fourth, we failed to classify our analysis according to different individual characteristics (eg, age or sex) of the victims; however, this would not bias our main findings but simply modify our estimates in different subgroups.

In summary, this time-series study provides scientific evidence suggesting that the criminalization of drunk driving since May 1, 2011, may have led to moderate reductions in RTIs in Guangzhou, China. This encouraging experience in China may also be helpful to other countries coping with road-safety problems. Further research is still needed to confirm our main findings.
